# Development of a resilience scale for oldest-old age (RSO)

**DOI:** 10.1186/s12877-021-02036-w

**Published:** 2021-03-10

**Authors:** Eiki Akatsuka, Etsuko Tadaka

**Affiliations:** Department of Community Health Nursing, Graduate School of Medicine, Yokohama City University, 3-9 Fukuura, Kanazawa-ku, Yokohama, Kanagawa 221-0825 Japan

**Keywords:** Adaptation to aging, Resilience, Oldest-old, Scale development

## Abstract

**Background:**

Globally, the population of oldest-old (those aged ≥80 years) is rapidly growing. This change is likely to have a deep impact on societies. Resilience is a key concept related to facilitating adaptation, and can be applied, to aging-related change and losses, as well as promoting health and well-being in this population. However, no existing scales have been developed to measure resilience among oldest-old people. To address this, we developed a resilience scale for oldest-old age (RSO), and examined its reliability and validity.

**Methods:**

The RSO is a self-administered questionnaire developed via a literature review, interviews with oldest-old individuals, and interviews with experts. The survey included 3000 community-dwelling oldest-old people who were recruited via random sampling in Yokohama city, Japan. Construct validity was determined using confirmatory factor analysis. Internal consistency was calculated using Cronbach’s alpha. The revised Philadelphia Geriatric Center Morale Scale (PGC) and the self-anchoring scale to measure the feeling that life is worth living (SAS-WL) were used to assess the criterion-related validity of the RSO.

**Results:**

We received 1283 valid participant responses. Confirmatory factor analysis identified nine items from one factor of the RSO with a goodness of fit index of 0.979, adjusted goodness of fit index of 0.963, comparative fit index of 0.973, and root mean square error of approximation of 0.049. Cronbach’s alpha was 0.800. The total RSO score was positively correlated with the PGC (r = .492, *p* < 0.001) and the SAS-WL (r = .559, p < 0.001).

**Conclusions:**

The RSO demonstrated adequate reliability and validity for assessing individual resilience among oldest-old people. Thus, the scale is potentially useful for promoting health and well-being in oldest-old age.

**Supplementary Information:**

The online version contains supplementary material available at 10.1186/s12877-021-02036-w.

## Background

The population of oldest-old (those aged ≥80 years) is rapidly growing. Worldwide, the number of oldest-old people is predicted to increase by more than three times between 2015 and 2050, from 126.5 million to 446.6 million [1]. In Japan, the proportion of the oldest-old in the total population is estimated to rise from 8.7% in 2018 to 15.8% in 2050 [2]. By 2050, one in seven Japanese people will be aged 80 years or older. With the increasing length of life and rising older population, there are concerns about significant increases in medical and long-term care costs in Japan [3]. Although improved life expectancy is a positive outcome, it gives rise to a new issue of how to ensure health and well-being among the oldest-old population.

Older people frequently experience aging-related changes and losses, such as declines in health and functioning, increased chronic illnesses and comorbidities, reduced independence, death of spouse/friends, and loss of social networks [4, 5]. Many previous studies [6–8] focused on preventing or delaying these changes and losses in older age. However, the oldest-old people have difficulty avoiding the impact of problems that increase with age, and adaptation to such changes and losses is critically important for well-being in later life [9–11]. In oldest-old age, prevention of aging-related changes or losses and adaptation to these aspects in their later life are essential to promoting health and well-being.

Resilience is a broad construct that involves concepts of adaptation. Although consensus around the definition of resilience is lacking, many authors have described resilience as an individual characteristic that allows people to cope with stress and adapt to threats [12, 13], or a dynamic process of adaptation [5, 14]. This concept includes multiple resources for adaptation, such as psychological resources (e.g., self-esteem, coping ability), health resources (e.g., health promotion, physical activity), and sociocultural resources (e.g., social networks, social capital). Resilience among older people has been related to health outcomes such as survival [15–17], independence [18], self-rated health [19], and depression [20, 21]. A review focused on resilience in oldest-old age [22] suggested that the conceptualization of resilience is the process of interaction between surrounding adversity, resource availability and mobilization, and positive outcomes. That review also found that to enable good adaptation in oldest-old age, it is important to focus on resilience, especially multiple individual resources such as psychosocial resources, valued activities, life experience, and spirituality. These findings suggest that resilience is an important concept in adapting to aging-related changes and losses and promoting health and well-being among the oldest-old population. However, no scale for evaluating resilience among the oldest-old is currently available. A search of the literature clarified that although measures exist for assessing psychological resilience in adults [12, 13, 23], along with broader resilience scales for adults [24] and older adults [25, 26], there is no concept or scale focused on adaptation to aging-related changes and losses that targets oldest-old people. It has been suggested that the resilience of oldest-old people differs from that of younger people in terms of constructs, correlates, and consequences [22, 27]. First, in terms of constructs, the resilience of the younger generation is characterized by personal characteristics, traits, and skills used to confront threats, whereas the resilience of the oldest-old old includes their life experiences, wisdom, and meaning in life. Second, in terms of correlates, the resilience of the younger generation includes resilience against age-neutral adversities, such as depression, anxiety, and traumatic experiences, whereas the resilience of oldest-old age includes resilience against age-specific stresses, such as function loss, physical health challenges, and diminished social networks due to aging. Third, in terms of consequences, the resilience of the younger generation focuses on the goals of recovery from illness and health promotion, while the resilience of the oldest-old focuses on maintaining independence and competency, such as comfort in daily life and a sense of one’s own value. Therefore, there is a gap in terms of a tool to assess resilience among this population.

This study aimed to develop a framework for a resilience scale for oldest-old age (RSO), and present findings on the psychometric properties of the scale. The overarching goal was to promote health and well-being among oldest-old people.

## Methods

### Phase 1: developing the RSO

First, we developed a pool of items. From the perspective of promoting health and well-being in oldest-old age, PubMed (1946–2019) and Ichushi-Web (1970–2019) were searched for articles about the process of adaptation to aging and the resilience among oldest-old people using specific keywords: resilience, adaptation, aging-related change, aging-related loss, oldest-old, fourth age, very old, and scale. This identified 13 article [12–14, 22–31].

Based on this review, we defined resilience as multiple individual resources for an adaptation process to aging-related changes and losses among oldest-old people. In accordance with this definition, the item pool includes a broader aspect of resources for adaptation to aging-related changes and losses such as psychological resources, health resources, and sociocultural resources. With reference to previous studies, a pool of items was developed based on: 1) the degree to which the given item reflected the definition of resilience; 2) the clarity of logic, meaning, and readability of the given item for oldest-old people; and 3) the practical usefulness of item. The initial total number of items was 45, of which 34 were derived from existing scales and 11 were created by referring to the literature and the researchers’ clinical experience. The draft item pool was reviewed and modified several times by us and several researchers, and reduced to 30 items that were focused on multiple resources for the process of adaptation to aging-related changes and losses in later life.

Next, the item pool was reviewed by seven oldest-old persons as primary reviewers, followed by three professionals and three researchers as secondary reviewers. This aimed to assess the content validity, face validity, and practical usefulness of the items for oldest-old people administered via interviews or questionnaires. The seven oldest-old reviewers lived in the community and were aged 80–89 years. The professional reviewer group included a nurse certified in visiting nursing, a public health nurse from a health center, and a care manager. The researchers in the review group were two professors and one assistant professor from the department of community health nursing. Before the survey, we provided a definition of resilience to participants, both in writing and verbally, as follows: 1) To the oldest-old old, we defined resilience as “the resources used to adapt flexibly to physical, emotional, and social discomforts and hardships associated with aging”; 2) To experts and researchers, we defined resilience as “the multiple individual resources required to adapt to aging-related changes and losses, such as physical, emotional, and social discomforts and hardships among oldest-old people”. Three main points were modified following the review: expressing the subject clearly, application to oldest-old people, and selection of words that better assessed resilience in double-barreled questions. The wording of each item was revised according to reviewers’ recommendations. Consequently, we initially excluded items assessed as “not important” by more than one reviewer. As a result, the modified RSO was refined to 20 items. In addition, we hypothesized a single-factor structure according to the definition of resilience.

### Phase 2: validating the RSO

#### Study participants

Study participants were 3000 community-dwelling individuals aged ≥80 years residing in Yokohama city, Japan. Participants were extracted using age classification stratified random sampling from Yokohama City Government via the Resident Registration System. The reasons for selecting Yokohama City were: 1) it is the largest government-designated city in Japan; and 2) it is a large city where rapid societal aging (including an increase in the oldest-old population) is estimated in Japan. Yokohama City is a government-designated city that includes 18 wards and has a population of 3.7 million people [32]. The percentage of the population in old-age is 24.3%, and that of the oldest-old is 7.3% [32].

Data were collected from September 20 to October 31, 2019. We mailed informed consent letters and questionnaires to selected participants. Each participant was invited to voluntarily complete the self-administered anonymous questionnaire. In the questionnaire, we asked participants to self-report as thoroughly and as honestly as possible, asked them to reflect on their thoughts, even if it was difficult to complete the questionnaire, and told them that family members were permitted to help them respond to the questionnaire or complete it on their behalf. Of the potential participants, 1363 (45.4%) responded, and 1283 (42.8%) questionnaires with valid responses were available for the analysis.

#### Measures

Participants’ demographic data included: age, sex, marital status, living status, residence status, certification for long-term care need under the Long-Term Care Insurance system in Japan (Support need level 1 and 2 and Care need levels 1 to 5; a larger number indicates a more severe level), and number of diseases currently under treatment.

Participants were asked to complete the modified 20-item version of the RSO. Each item was assessed on a 4-point Likert-type scale: 0 = disagree, 1 = disagree somewhat, 2 = agree somewhat, and 3 = agree. Missing data were treated as follows. One missing value was substituted with the average value for the other items [33, 34]. If more than one item was missing, the response to that questionnaire was considered invalid and no total score was calculated.

To assess the concurrent validity, the researchers used two measures. First, we used the revised Philadelphia Geriatric Center Morale Scale (PGC) [35], which was available in a Japanese version [36]. The PGC comprises 17 items belonging to three factors: agitation, attitude toward own aging, and lonely dissatisfaction. Items are scored from 0 to 1 (range 0–17). High PGC scores indicate a high level of subjective well-being in later life. The Cronbach’s alpha for the PGC was 0.837 in the present study. The researchers hypothesized the RSO would positively correlate with subject well-being in later life.

The second measure was the Self-Anchoring Scale to Measure the Feeling that Life is Worth Living (SAS-WL) [37], which is based on the ladder method of the Self-Anchoring Striving Scale [38]. Central concepts of the “feeling that life is worth living” are the sense of motivation to achieve purpose, usefulness, and responsibility. The SAS-WL requires individual participants to subjectively define 0 (Not at all) to 10 (Enough) choices on a ladder device. High SAS-WL scores indicate a high level of the feeling that life is worth living. The test-retest reliability and construct validity was confirmed with comparative research [37]. In this study, we expected that the higher RSO scores correlate with higher scores with the feeling that life is worth living.

#### Statistical analysis

Item analyses were conducted to ensure that only pertinent, functional, and internally consistent items were included. The criteria for item analysis included rates of difficulty (non-respondents ≥5%), skewness or kurtosis (absolute values < 1.0), correlations of each item (correlation coefficient > 0.7), good-poor analysis (no significant differences between the highest- and lowest-scoring groups), and item-total analysis (correlation coefficient < 0.3).

After item analysis, the total sample (*n* = 1283) was randomly divided into two groups for cross-validation: group 1 (*n* = 642) for performing exploratory factor analysis (EFA) and group 2 (*n* = 641) for performing confirmatory factor analysis (CFA). The items remaining after item analysis were examined using exploratory factor analysis. The optimal number of factors was determined by sequentially using latent root criteria (eigenvalues > 1.0) and a scree plot. Item loadings needed to exceed 0.40. CFA was then conducted to verify the construct validity. The goodness-of-fit index (GFI), adjusted goodness-of-fit index (AGFI), comparative fit index (CFI), and root-mean-square error of approximation (RMSEA) were used to evaluate the data model fit. The model was accepted if the GFI, AGFI, and CFI indices were ≥ 0.90 and the RMSEA was ≤0.05. Cronbach’s alpha was used to evaluate the internal consistency of the RSO, with a value of ≥0.70 considered adequate. Also, criteria-related validity was examined using the PGC and SAS-WL. Furthermore, known-group validity was examined using a t-test (group without long-term care need vs. with long-term care need).

All analyses were conducted with IBM SPSS Statistics Version 23.0 and Amos 23.0.

## Results

### Participant characteristics

Table 1 shows participants’ characteristics. The mean age was 84.7 years; 44.6% were female, 53.9% were married, 25.9% were living alone, and 29.0% were certificated for long-term care need. The mean number of diseases currently under treatment was 1.8.

**Table 1:** Participants’ demographic characteristics

		(*n* = 1283)	
		Number or	% or
		Mean ± SD	(Range)
Age, years		84.7 ± 4.1	(80–105)
	80–84	724	56.5
	85–89	375	29.2
	90–95	123	9.6
	95–99	37	2.8
	≥100	2	0.2
	Missing	22	1.7
Sex	Female	572	44.6
	Male	691	53.9
	Missing	20	1.6
Marital status	Married	692	53.9
	Divorced/widowed	541	42.2
	Unmarried	24	1.9
	Missing	26	2.0
Living status	Living with spouse	436	34.0
	Living alone	332	25.9
	Living with children	221	17.3
	Living with spouse and children	143	11.1
	Living with children and grandchild	64	5.0
	Others	67	5.2
	Missing	19	1.5
Resident status	Home	1073	83.6
	Others	172	13.4
	Missing	38	3.0
Certification for long-term care insurance	No	830	64.7
	Yes	372	29.0
	Support need levels 1 and 2	168	13.1
	Care need levels 1 and 2	112	8.8
	Care need levels 3–5	88	6.8
	Missing	81	6.3
Number of diseases currently under treatment		1.8 ± 1.2	(0–7.0)
	Hypertension	571	44.5
	Arthritis, osteoporosis	307	24.7
	Cataract, glaucoma	281	21.9
	Diabetes, hyperlipidemia, hyperuricemia	280	21.8
	Myocardial infarction, angina	142	11.1
	Others	268	20.9
	Missing	39	3.0

*SD* standard deviation

### Item analysis

The results of the item analysis are shown in Table 2. First, nine items (items 1, 4, 5, 6, 7, 9, 12, 15, and 17) did not meet the criterion of normality based on kurtosis and skewness. Second, two items (items 18 and 19) did not meet the criterion of exhibiting correlations between item 18 and 19 (correlation coefficients > 0.70). Third, four items (item 5, 6, 11, and 12) did not meet the criterion of item-total correlations. Based on these results, 11 items (items 1, 4, 5, 6, 7, 9, 11, 12, 15, 17, and 18) were excluded to ensure the pertinence, functionality, and internal consistency of items, and nine items (items 2, 3, 8, 10, 13, 14, 16, 19, and 20) were used for the factor analysis.

**Table 2:**
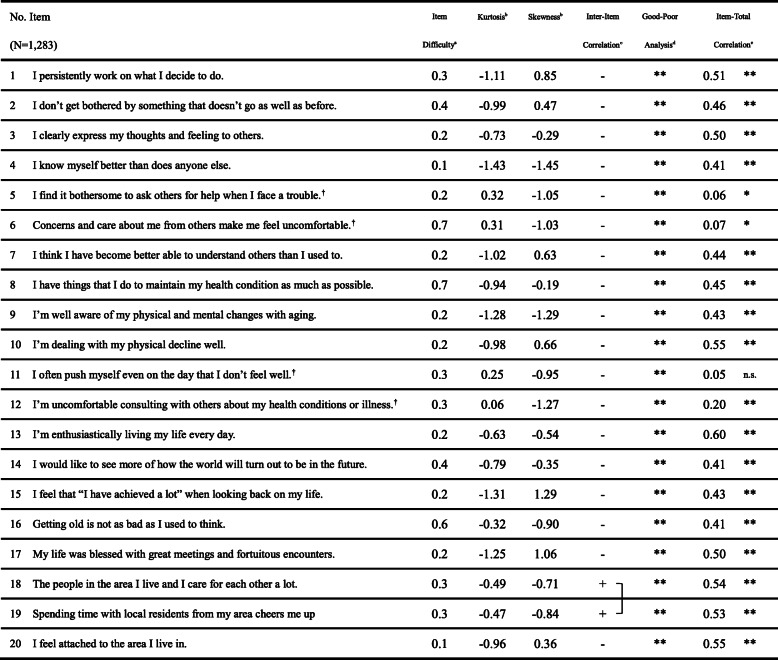
Item analysis of the “resilience scale for oldest-old age”

^†^:Reversed item,×: Excluded item, **: *p* < 0.01, *: *p* < 0.05, n.s.: not significant

Exclusion criteria for the item analysis

^a^: The percentage of no answer over 5% of the sample

^b^: Absolute values of skewness or kurtosis < 1.0

^c^: Correlation over 0.7

^d^: Difference of the average score between the highest-scoring group and lowest-scoring group was not significant (*p* ≥ 0.05)

^e^: The correlation coefficient between the item and the total of all the items (with the exception of that item) was less than 0.3

### Factor structure

**Fig. 1:**
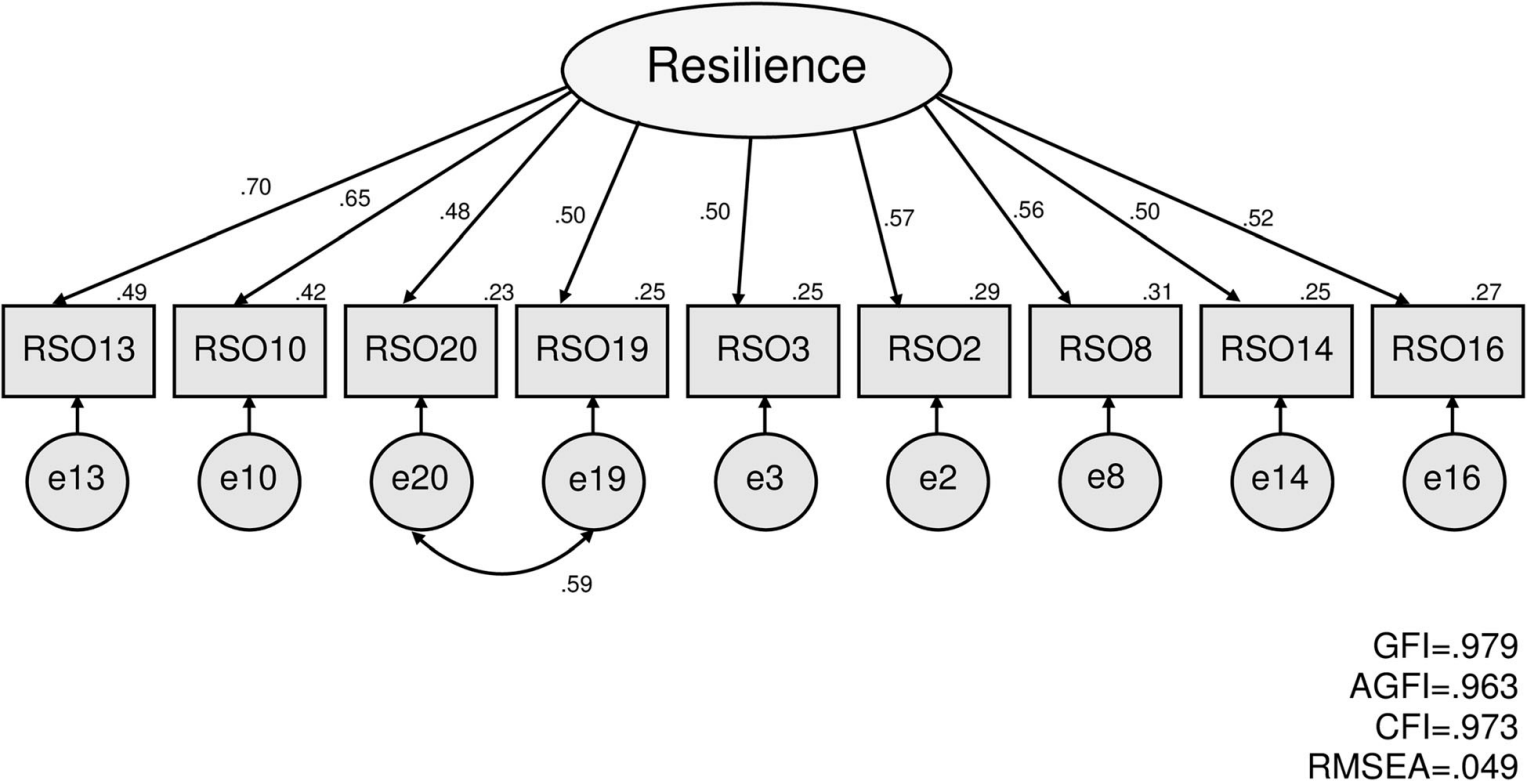
Confirmatory factor analysis of the “Resilience scale for oldest-old age” (final version)

The latent root criteria (eigenvalues > 1.0) and the scree plot indicated either a one or two factor model because of the way the slope leveled off twice. In the EFA (Table 3), the first factor together explained most of the variability in the original nine items, which suggested the RSO had good conceptual consistency (31.7% of the variance explained, Cronbach’s alpha 0.798). In the CFA, the initial model showed that the GFI = 0.905, AGFI = 0.842, CFI = 0.807, and RMSEA = 0.127, which did not represent a good data-model fit. The model fit was improved after modifying the model according to modification indices, and adding error correlations for items 19 and 20 (GFI = 0.979, AGFI = 0.963, CFI = 0.973, RMSEA = 0.049), which satisfied the appropriate criteria in all subjects (Fig. 1). We found an error correlation between items 19 and 20; item 19 measured the feeling of being empowered through person-to-person relationships, whereas item 20 measured the sense of attachment to one’s own community as a whole, and not that which is limited to person-to-person relationships. Hence, we hypothesized that items 19 and 20 measured independent resources for adaptation to aging. However, items 19 and 20 both measured the feeling of whether the necessary support and community relationships were provided. Thus, the positive correlation between the errors was considered to reflect the more general perception of their community. Therefore, the final version of the RSO was judged as having nine items with a single factor structure.

**Table 3:** Exploratory factor analysis of the “Resilience scale for oldest-old age”

No.	(*N* = 642)	
	Item	Loading
13	I’m enthusiastically living my life every day.	0.760
10	I’m dealing with my physical decline well.	0.618
20	I feel attached to the area I live in.	0.563
19	Spending time with local residents from my area cheers me up	0.549
3	I clearly express my thoughts and feeling to others.	0.548
2	I don’t get bothered by something that doesn’t go as well as before.	0.522
8	I have things that I do to maintain my health condition as much as possible.	0.495
14	I would like to see more of how the world will turn out to be in the future.	0.481
16	Getting old is not as bad as I used to think.	0.476
Eigenvalue		3.508
Cronbach’s alpha		0.798

Principal factor analysis with non-rotation

### Internal consistency and validity

The Cronbach’s alpha coefficient was 0.800 for the final version of the RSO, showing the scale had sufficient internal consistency. The final version of the RSO was positively correlated with both the PGC (r = 0.492; *p* < 0.001) and the SAS-WL (r = 0.559; p < 0.001) (Table 4). Furthermore, the mean RSO score was significantly higher in the group without long-term care need than in the group with long-term care need (19.0, SD, 4.6; 16.8, SD: 5.8; p < 0.001).

**Table 4:** Internal consistency and criteria-related validity

Internal Consistency (*n* = 1283)	Cronbach’s alpha	0.800
Criteria-related validity		
PGC (*n* = 1144)	R^a^	0.492***
SAS-WL (*n* = 1224)	R^a^	0.559***

^a^: Pearson’s correlation, ***: *p* < 0.001

Notes

*PGC* Revised Philadelphia Geriatric Center Morale Scale Japanese version, *SAS-WL* Self-Anchoring Scale to Measure the Feeling that Life is Worth Living

## Discussion

### Representativeness of study participants

The participants in this study were considered to be representative of community-dwelling oldest-old people in Japan. We conducted a self-administered anonymous questionnaire survey with random sampling, and the response rate in this study was sufficient (45.4%). The percentages of participants aged 80–89 years, 90–99 years, and ≥ 100 years were 85.7, 12.4, and 0.2%, respectively; this was similar to the percentages for these age groups in Yokohama City (82.0, 17.3, and 0.5%, respectively) [32], and in Japan (79.8, 19.6, and 0.6%, respectively) [39].

### Originality of the RSO

The originality of this research was the development of a new RSO for use among the oldest-old to support adapting to aging-related changes and losses and promoting health and well-being (Additional files 1, and 2). The results showed the RSO had adequate reliability and validity. The CFA model verified the factor validity (GFI = 0.979, AGFI = 0.963, CFI = 0.973, RMSEA = 0.049) and factor correctness of a set of nine observed variables within one factor. In terms of the reliability of the RSO, the Cronbach’s alpha indicated the scale had sufficient internal consistency. The criterion-related validity was 0.492 between the RSO and PGC, and 0.559 between the RSO and SAS-WL. Therefore, the RSO developed through this research was judged to be a sufficient, reliable, and valid scale that is capable of effectively assessing the resilience for adapting to aging-related changes and losses among oldest-old people.

There are similarities and differences between the RSO and previous resilience scales [12, 13, 23–26]. All of the previous scales highlighted the personally traits or processes used to moderate stress and adapt to threats. However, the RSO differs in the core construct and target population. While the core constructs of resilience in the previous measure focused on individual characteristics and traits that help individuals confront age-neutral adversity, the core constructs of resilience in the RSO focus on multiple individual resources for adapting to age-related changes and losses in oldest-old age individuals. In addition, the target of the RSO is the community-dwelling oldest-old population. Hayman [22] noted that a resilience scale for oldest-old people is necessary because the resilience of this group differs from constructs and challenges among younger people. Oldest-old people may draw on their life experiences and wisdom to adapt to the changes and losses that come with aging, but no scale has yet been developed to measure resilience of the oldest-old. The RSO measures resilience in adaptation to aging-related changes and losses among this population. The scale will make it possible to promote health and well-being among oldest-old people by assessing resilience as a multiple individual resource for the process of adaptation to aging-related change and losses.

### Usability of the RSO

Use of the RSO for oldest-old individuals will support professionals to assess the resilience among this group and inform an individualized support approach. In addition, by explaining professional evaluations to individuals, the oldest-old person can be made aware of their own strengths or weaknesses related to adaptation to aging. For example, an oldest-old individual with a high RSO score can adapt to aging-related difficulties and maintain well-being in later life. Conversely, oldest-old individuals with a low RSO score are more vulnerable to these difficulties, and may require help to deal with their difficulties and adapt to the aging process in daily life. However, further research is needed to develop effective practices or self-care methods to promote resilience among the oldest-old.

In addition, use of the RSO in communities or facilities will allow the resilience of the oldest-old population in those settings to be evaluated. Moreover, a survey using the RSO jointly with other communities can help to understand the strengths and weaknesses of one community from comparisons with other communities. Specifically, the RSO could be used by government organizations to measure the resilience of oldest-old people in their communities through resident surveys and project evaluations, and may provide knowledge necessary for community development. For example, if one community has a higher total score on the scale than other communities, this would suggest that oldest-old people in this community have abundant resources to utilize their strengths and adapt to aging, which may serve as a good model for the development of other communities. Conversely, if a community has a lower score on the scale than other communities, it is possible that the oldest-old people living in that community are not adapting well to the changes and losses associated with aging, and are seeking the resources necessary to adapt. This may help to identify and develop the resources needed for oldest-old people within a community. Previous research suggests that resilience is enhanced by the environment and social context in a community [22, 27, 40]. Therefore, RSO scores may be useful to inform program, system, and community-level strategies aimed at promoting health and well-being among oldest-old people and communities. Furthermore, use of the RSO may show to people who consider older people to simply be a burden that their life experience and age has in fact enhanced their ability to bounce back and cope with life stressors. Effective and evidence-based programs and systems need to be developed to popularize the RSO to support adapting to aging and promote aging-well among oldest-old people in communities.

### Limitations

The present study had a few limitations. First, as the study design was cross-sectional, it could not clarify any association between the RSO and health outcomes among oldest-old people. Therefore, a study with a prospective design is needed to determine the scale’s predictive validity. Second, although a major city in Japan was used as the study setting, it would be useful to examine data for other community or country contexts. According to previous studies [22, 27], resilience is not context-free, but rather is highly dependent on situational environment and community characteristics or culture. Further studies are needed to investigate the construct validity of the RSO in other communities or countries that may have different characteristics from the present participants. Third, this study did not investigate the differences between the present and previous scales of resilience. Further studies are needed to examine the discriminant validity of RSO.

## Conclusion

The RSO is a novel instrument with good psychometric properties for assessing resilience for the purpose of promoting health and well-being for oldest-old people. The RSO has potential utility for promoting practices, interventions, and health policies to ensure aging-well in oldest-old age.

## Supplementary Information

**Additional file 1.** English version of the final RSO.

**Additional file 2.** Japanese version of the final RSO.

## Data Availability

The datasets generated and analyzed during the current study are not publicly available because the Ethical Guidelines for Epidemiological Research by the Japanese Government and the National Basic Resident Registration System administered by the Ministry of Internal Affairs and Communications in Japan prohibit researchers from providing their research data to other third-party individuals but are available from the corresponding author on reasonable request.

## Abbreviations

AGFI: Adjusted goodness-of-fit index
CFA: Confirmatory factor analysis
CFI: Comparative fit index
GFI: Goodness-of-fit index
PGC: The revised Philadelphia Geriatric Center Morale Scale
RMSEA: Root-mean-square error of approximation
RSO: The resilience scale for oldest-old age
SAS-WL: The self-anchoring scale to measure the feeling that life is worth living
