# Supplementary material for: Development of a resilience scale for oldest-old age (RSO)
Source: BMC Geriatr. 2021 Mar 10;21:174. doi: 10.1186/s12877-021-02036-w (PMC7944912; doi:10.1186/s12877-021-02036-w)
Supplement: Supplementary file 1 — Additional file 1. English version of the final RSO. [file 12877_2021_2036_MOESM1_ESM.docx]

**Additional file 1: English version of the final RSO**

**RSO: Resilience Scale for Oldest-old age**

| No. | Item | Disagree | Disagree somewhat | Agree somewhat | Agree |
| --- | --- | --- | --- | --- | --- |
| 1 | I’m enthusiastically living my life every day. | 0 | 1 | 2 | 3 |
| 2 | I’m dealing with my physical decline well. | 0 | 1 | 2 | 3 |
| 3 | I feel attached to the area I live in. | 0 | 1 | 2 | 3 |
| 4 | Spending time with local residents from  my area cheers me up. | 0 | 1 | 2 | 3 |
| 5 | I clearly express my thoughts and feeling  to others. | 0 | 1 | 2 | 3 |
| 6 | I don’t get bothered by something that  doesn’t go as well as before. | 0 | 1 | 2 | 3 |
| 7 | I have things that I do to maintain my  health condition as much as possible. | 0 | 1 | 2 | 3 |
| 8 | I would like to see more of how the world  will turn out to be in the future. | 0 | 1 | 2 | 3 |
| 9 | Getting old is not as bad as I used to think. | 0 | 1 | 2 | 3 |

Akatsuka E, Tadaka E: Development of a resilience scale for oldest-old age (RSO), BMC Geriatrics,2021.
