# Supplementary material for: Development of a resilience scale for oldest-old age (RSO)
Source: BMC Geriatr. 2021 Mar 10;21:174. doi: 10.1186/s12877-021-02036-w (PMC7944912; doi:10.1186/s12877-021-02036-w)
Supplement: Supplementary file 2 — Additional file 2. Japanese version of the final RSO. [file 12877_2021_2036_MOESM2_ESM.docx]

Additional file 2: Japanese version of the final RSO


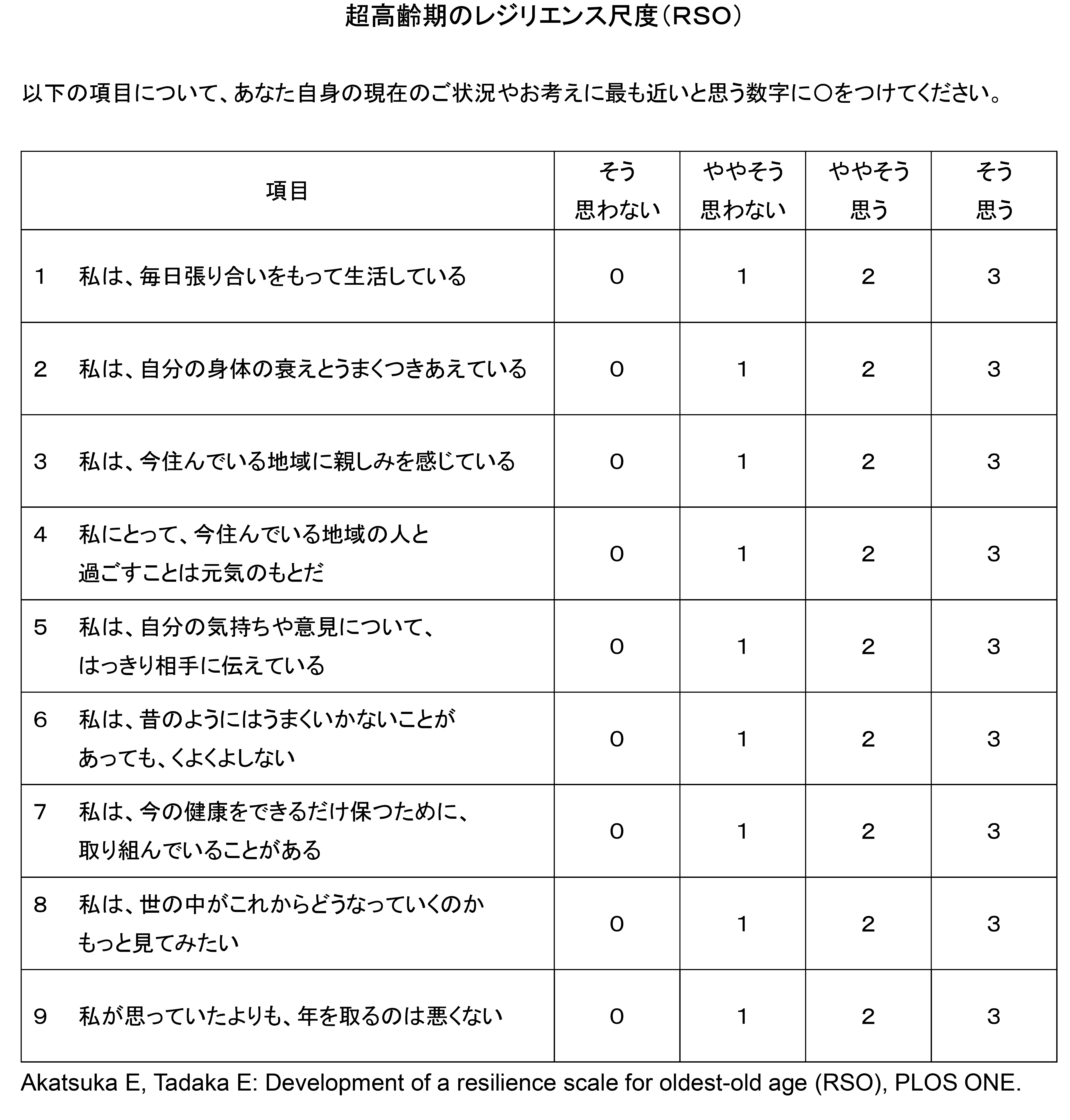


Akatsuka E, Tadaka E: Development of a resilience scale for oldest-old age (RSO), BMC Geriatrics,2021.
